# COVID-19 Vaccine Uptake among Younger Women in Rural Australia

**DOI:** 10.3390/vaccines10010026

**Published:** 2021-12-27

**Authors:** Jessica Carter, Shannon Rutherford, Erika Borkoles

**Affiliations:** School of Medicine and Dentistry, Griffith University, 1 Parklands Dr., Southport, QLD 4215, Australia; s.rutherford@griffith.edu.au (S.R.); e.borkoles@griffith.edu.au (E.B.)

**Keywords:** COVID-19, vaccination, vaccine uptake, vaccine hesitancy, vaccine literacy, rural health, women’s health, mixed methods

## Abstract

Vaccine uptake in younger Australian women living in rural and regional communities is poorly understood. This research explored factors affecting their decision making in the context of social determinants of health. A mixed methods design applying an explanatory sequential approach commenced with an online questionnaire followed by in-depth interviews with a sample of the same participants. The majority (56%) of participants indicated a positive intention to be vaccinated against COVID-19, but a substantially high proportion (44%) were uncertain or had no intention to be vaccinated. Significant factors affecting vaccine uptake included inadequate and sometimes misleading information leading to poor perceptions of vaccine safety. The personal benefits of vaccination—such as reduced social restrictions and increased mobility—were perceived more positively than health benefits. Additionally, access issues created a structural barrier affecting uptake among those with positive or uncertain vaccination intentions. Understanding factors affecting vaccine uptake allows for more targeted, equitable and effective vaccination campaigns, essential given the importance of widespread COVID-19 vaccination coverage for public health. The population insights emerging from the study hold lessons and relevance for rural and female populations globally.

## 1. Introduction

The novel coronavirus disease known as COVID-19 has caused significant health, social and economic challenges globally. For much of the crisis, vaccination has been framed as the central solution to managing the pandemic. Vaccinations are among the world’s most impactful public health interventions, and the rapid development of COVID-19 vaccines is a pandemic success story. However, the true measure of success will be determined by high rates of vaccine uptake globally and locally.

Vaccine uptake is the proportion of eligible individuals who receive a specific vaccine in a specific period [1]. Vaccine uptake is not only a public health objective, but also a process of public decision-making informed by psychological, sociocultural, and political processes [2]. There are many approaches to measure vaccine uptake across populations. This study used an existing taxonomy of determinants to conceptualize factors affecting vaccine uptake—access (the ability to be reached by vaccines), affordability (the ability to afford vaccination in terms of time and financial cost), awareness (the ability to understand the need for and availability of vaccines), acceptance (the degree to which individuals accept, question, delay or refuse vaccination) and activation (the degree to which individuals are nudged towards vaccination through rewards or punishments) [3]. Furthermore, factors shaping the process of vaccine uptake vary according to the contextual circumstances and social determinants of each population, vaccine and disease [4,5,6].

Achieving COVID-19 vaccination coverage targets therefore depends on understanding the needs and circumstances of specific populations so that vaccine campaigns can address the unique factors shaping uptake at a population level. Globally, research into perceptions towards COVID-19 vaccination has found that among general adults, women and younger age groups are among those populations who have indicated a lower willingness to be vaccinated against COVID-19 [7,8,9,10,11]. Similarly, in Australia, research exploring COVID-19 vaccine uptake found that younger Australian women (in the 30–39 and 35–44 cohorts) demonstrated higher levels of concern for COVID-19 vaccine safety than the rest of the population [12,13]. This is significant because women often carry health care responsibilities for their households [14], meaning they are important decision-makers whose perspectives on public health interventions, such as vaccination, may influence not only their own health behavior but also their partners and children.

Research identifying concerns about COVID-19 vaccination among younger Australian women found that social context and disadvantage played a parallel role in shaping vaccine uptake [12,15]. Regional and rural Australians experience greater disadvantage than their urban counterparts [16]. In rural Australia, access to and usage of health services is lower than in urban centers [17,18,19] which can lead to the lower life expectancy and higher rates of injury and disease experienced by regional and rural residents [20]. Access issues can also be compounded by poverty, and rural and regional parts of Australia have lower socio-economic levels than urban areas [17]. The intersection of the issues of access and higher rates of injury and disease in rural and regional geographies present additional challenges for COVID-19 vaccine uptake. Yet, research into vaccine uptake in rural and regional Australian populations is limited.

Australia’s COVID-19 vaccine rollout has been plagued by procurement and distribution issues [21], leading to unpredictable and inconsistent access. There is a risk that underlying concerns around vaccine safety among younger women could compound with challenges around overall access in regional and rural areas leading to lower vaccine uptake. If this were to occur, the implications would be significant, since the poorer health care access and lower health outcomes in regional and rural areas would create greater health system challenges if a COVID-19 outbreak were to occur, leaving under-vaccinated populations, such as younger women, particularly vulnerable. Therefore, this mixed methods study examined the factors affecting vaccine uptake for women living in rural and regional areas of Australia aged between 30–44.

## 2. Materials and Methods

### 2.1. Study Design and Procedures

The study used a mixed methods approach with an explanatory sequential design [22]. The research commenced with a quantitative, cross-sectional questionnaire. The findings of this Phase 1 informed the qualitative Phase 2 of semi-structured, in-depth interviews with participants who volunteered for the study in Phase 1.

Women aged 30–44 were recruited for Phase 1 using a convenience sampling method. The online questionnaire was promoted through social media channels targeting Australian women living in rural or regional areas, and snowball sampling was used to expand the reach. Online questionnaire participants had the option of choosing to indicate their interest in participating in Phase 2, an in-depth interview, and those candidates were screened through a purposive sampling method to ensure a diverse range of interview participants were chosen. Informed consent was collected electronically, for both instruments, and data protection and privacy measures were taken to protect participant confidentiality.

Eligibility for both phases was based on self-identification with the targeted gender and age, along with criteria around rural location, and that they had not received a COVID-19 vaccination at the time of participating. The criterion to include only those who had not received a COVID-19 vaccination was to ensure a consistent measurement of vaccination intention before any vaccination behavior occurred. While vaccine intention does not necessarily equate to vaccine uptake, there is a demonstrated association between both higher hesitancy and lower uptake or lower hesitancy and higher uptake [23]. The definition of the rural and regional setting was based on the Australian Bureau of Statistic’s application of the Australian Statistics Geography Standard—Remoteness Areas (ASGS–RA); postcodes of questionnaire respondents were manually screened and coded against the ASGS–RA categories of Inner Regional, Outer Regional, Remote and Very Remote [24] to ensure they met the setting criterion. No other exclusion criteria were applied.

Responses to the confidential online questionnaire were collected over approximately eight weeks, from 1 June to 24 July 2021. The questionnaire was piloted with a small group representing 5% of the sample before launching it publicly. The target minimum responses were 90, assuming a 90% confidence level and a 5% margin of error.

In-depth interviews were conducted over approximately one week, from 30 July until 8 August 2021. Each in-depth interview took up to one hour and was transcribed automatically and checked manually. The transcripts were then analyzed to ensure that data saturation had been reached. The target for interviews was 10, based on approximately 10% of the questionnaire sample and saturation of data evaluations.

### 2.2. Questionnaire Measures

The questionnaire used in Phase 1 consisted of 31 questions across four sections, covering the participants’ sociodemographic characteristics, their information sources and vaccine literacy, perceptions of vaccine risks, benefits and trust, and vaccine access. The key sociodemographic characteristics examined included educational level, employment status, pregnancy status and number of children. The vaccine literacy questions measured functional vaccine literacy—defined as basic comprehension [25]—and interactive-critical vaccine literacy—defined as ability to critically analyze and apply meaning [25]—using a 4-point Likert scale validated for content and construct [26] and used twice with a general adult population on COVID-19 vaccine literacy [26,27]. The score was obtained from the mean value of the answers to each scale (ranging from 1 to 4), with a higher value corresponding to a higher vaccine literacy level. Perceptions of vaccine risks related to both COVID-19 and general vaccination were measured using three questions adapted from a previously tested survey [26,27] which included a measure of COVID-19 vaccination intention, the outcome variable for the study. Three questions focused on perceived benefits of vaccination, designed to reflect Australian Government messaging on positive outcomes of COVID-19 vaccination. Two questions focused on perceived trust in national and state/territory policy and political decision-making related to COVID-19 vaccination. One question was adapted from a global survey of COVID-19 vaccine intention [28], and measured practice of other preventive behaviors practiced, shown to have an association with vaccine acceptance [8]. Two questions examined vaccine access focused on COVID-19 vaccine and healthcare access in an Australian setting, known to be a factor affecting rural health outcomes [24].

### 2.3. In-Depth Interview Measures

In-depth interviews in Phase 2 were conducted using a topic guide developed for the purpose of the study, drawing on questions developed to measure COVID-19 vaccine intention [29]. Eight open questions examined similar themes from the questionnaire, including a deeper examination of the questionnaire’s outcome variable (intention to be vaccinated against COVID-19). Questions were also tailored based on the interviewee’s responses in the questionnaire.

### 2.4. Data Analyses

Statistical analyses of quantitative data collected in Phase 1 were performed using SPSS version 28 for Mac. Descriptive statistics were used for all the variables. Chi-square tests were used to measure the outcome variable’s association with all other nominal and ordinal variables since the data did not follow a normal distribution; a p value of less than 0.05 was used to determine statistical significance. Logistic regression analyses were conducted using the dependent variable—intention to be vaccinated against COVID-19—and independent variables which showed statistical significance in the chi-square tests. The internal consistency of the vaccine literacy scales was assessed through Cronbach’s alpha coefficient.

Qualitative analyses of Phase 2 interviews were conducted using a grounded theory analysis technique [30]. After identifying initial codes in the transcripts, focused coding was completed. Quotes from the transcripts that captured specific representations of each focused code were also identified. Data from Phases 1 and 2 were cross-referenced using the Pillar Integration Process [31] to identify key themes.

## 3. Results

### 3.1. Quantitative Results

#### 3.1.1. Population Characteristics

Eligible respondents (*n* = 90) had a median age of 36 years. Most respondents lived in Inner Regional locations (38%), followed by Outer Regional locations (33%). Most respondents were tertiary educated (72%) and employed (including healthcare professionals) (75%). Most had one or more children (62%) and a small portion of respondents (9%) were pregnant at the time of completing the questionnaire. All sociodemographic characteristics of the questionnaire respondents are reported in Table 1.

#### 3.1.2. Factors Affecting COVID-19 Vaccine Uptake

The outcome variable measured was participants’ COVID-19 vaccination intention. Vaccination intention was measured on a 3-point Likert scale of Yes, Uncertain and No, with a majority indicating positive acceptance (56%) followed by a combined total of Uncertain and No representing negative acceptance (44%).

Most respondents indicated that their preferred COVID-19 vaccine was Pfizer (72%) followed by those who indicated that they preferred not to be vaccinated at all (20%); other preferences accounted for 10%. Access was a noted issue, with the majority (57%) indicating that they were uncertain about or did not have nearby access to their preferred vaccine.

Many respondents indicated negative views of COVID-19 vaccine safety and efficacy, indicating they did not trust the vaccine’s safety (58%) or effectiveness (60%). Views towards general vaccination indicated a higher level of overall acceptance. The majority had previously received a vaccination for influenza (79%). Most respondents disagreed with the statements “I am not in favour of vaccines because they are unsafe” (84%) and “There is no need to vaccinate because natural immunity exists” (84%).

The main findings of the questionnaire around vaccine acceptance are reported in Table 2.

The information sources used most frequently by respondents were online news (52%), social media (46%), government websites (44%), television (43%) and family or friends (33%), health professionals (30%) and other online sources (20%). Respondents made use of multiple sources of information.

The mean score of functional vaccine literacy was 2.92, while the interactive-critical vaccine literacy score was 3.06, out of a maximum of 4. The vaccine literacy score has good internal consistency, with a Cronbach alpha coefficient reported of 0.80 for the functional scale and 0.70 for the interactive-critical scale [27]. In this study, the Cronbach alpha coefficient was 0.85 for the functional scale and 0.83 for the interactive-critical scale.

Two-thirds of respondents believed that getting a COVID-19 vaccine would enable them to travel safely within Australia (60%) while half believed that getting a COVID-19 vaccine would enable them to travel internationally again (53%).

Most respondents practiced preventive health behaviors of washing hands regularly with soap and water (96%) or covering mouth and nose when coughing or sneezing (92%). Other popular measures were avoiding close contact with anyone who has a fever or cough (84%), staying at least 1.5 meters away from other people (72%); cleaning or disinfecting surfaces (65%) and wearing a face mask (60%).

A Chi-square Test of Independence was performed to assess the relationship between the outcome variable—intention to be vaccinated against COVID-19—and each independent variable. The most statistically significant associations between the intention to be vaccinated against COVID-19 and other variables were perceptions towards COVID-19 vaccination for children (*p* < 0.001) and perceptions towards the benefits of vaccination—easier domestic travel (*p* < 0.001), reduced social restrictions (*p* < 0.001), or easier international travel (*p* = 0.004). Among information sources, use of online news as a primary information source had a significant association with the outcome variable (*p* = 0.004). Among preventive behaviors, wearing a mask had a significant association with the outcome variable (*p* = 0.012). There was no significant association identified between the outcome variable and demographic factors, vaccine literacy or trust in government. The complete list of significant associations with the outcome variable identified through the Chi-square Test of Independence are listed in Table 3.

Logistic regression was performed to assess the impact of a group of predictor variables on the odds that respondents would report an intention to be vaccinated against COVID-19. Three models were tested, and one was statistically significant—this model was performed to assess the impact of Information Sources (television, online news, government websites, radio and newspaper) on intention to be vaccinated against COVID-19. The full model containing all predictors was statistically significant, χ^2^ (5, *n* = 79) = 17.36, *p* = 0.004, indicating that the model was able to distinguish between respondents who reported a positive intention versus those who had a negative or uncertain intention to be vaccinated against COVID-19. The model correctly classified 79% of the intention to be vaccinated against COVID-19. The Cox and Snell R Square of 0.197 shows that 20% of intention to be vaccinated against COVID-19 is explained by participants getting their information from television alone about the COVID-19 vaccine.

### 3.2. Qualitative Findings

#### 3.2.1. Population Characteristics

Interviewees (*n* = 10) had a median age of 36. Most interviewees lived in Inner Regional or Outer Regional locations, were tertiary-educated and employed (including healthcare professionals) and had one or more children. No interviewees had personally contracted the COVID-19 disease or travelled overseas since the Australian Government closed its borders in March 2020; the majority did not know anyone who had contracted COVID-19 (inside or outside Australia).

#### 3.2.2. Factors Affecting COVID-19 Vaccine Uptake

The interviews identified 26 factors affecting vaccine uptake, which were grouped into five thematic categories—information sources and vaccine literacy, vaccine acceptance, perceptions of trust and vaccination benefits, vaccine access, and vaccination intention.

In the category of information sources and vaccine literacy, factors affecting vaccine uptake were inadequate and sometimes misleading information leading to poor perceptions of vaccine safety. One factor affecting younger rural women’s vaccine uptake was the perception that available information on COVID-19 vaccines and disease did not meet the interviewees’ needs or answer their questions, leading to widespread confusion and uncertainty. For example, many interviewees indicated low trust in available information, characterized by ideas that available information was polarized, and agenda driven. Most interviewees stated that they didn’t trust news media representations of the COVID vaccine, regardless of whether the content was framed as positive or negative. Another important factor affecting vaccine uptake was being overwhelmed or confused by information. Some interviewees responded to this by avoiding information about the vaccination or disease as they found the topic caused anxiety. Other interviewees felt that the information they wanted was not readily available, and despite the surplus of information, their questions often remained unanswered. Interviewees felt that they needed more specific and clear information related to their circumstances, such as women who were pregnant or breastfeeding desiring more contextualized facts for their situation.

Factors relating to vaccine acceptance indicated that a significant barrier to uptake was concerns around COVID-19 vaccine safety, which was framed as an issue emerging from the speed of the vaccine’s development. Some interviewees had concerns about the long-term health effects of the COVID-19 vaccine being unknown, while others had specific concerns about vaccine ingredients. These concerns contrasted with overall acceptance of other vaccines.

Factors relating to perceptions of trust and vaccination benefits showed a widespread importance of the social and personal benefits of being vaccinated among younger rural women. Many were not personally concerned about catching COVID-19 in part because at the time interviews were conducted COVID-19 disease outbreaks in rural and regional Australia were rare. Enabling factors affecting COVID-19 vaccine were perceived social and personal benefits, which played a greater role in influencing vaccination intention than health benefits. Another factor that supported this was concern about the impact of social restrictions being more negative than the impact of the COVID-19 disease on interviewee’s lives. Factors that acted as barriers were low trust in government and the overall approach to the vaccine rollout—only a small group expressed trust in the government approach to managing the situation. Others were concerned about the vaccine rollout, seeing it as ‘trial and error’, and there were concerns that rural people were a lower priority than their urban counterparts.

Related to some of the concerns with the vaccine rollout, access emerged as a significant factor affecting younger rural women, particularly among those who wanted to be vaccinated, but had not yet done so. Many interviewees were unsure about how to be vaccinated, uncertain about their eligibility or unsure about where to go to book an appointment. Some had already tried to book a vaccine appointment but were unable to because they could not get an appointment (either due to eligibility or availability), or they were unable to access any vaccine (due to supply issues). Factors that limited access were logistical barriers that would affect how soon or whether they could be vaccinated. For example, many identified childcare as an issue, either while they receive a vaccine or if they experience side effects from the vaccine. Some interviewees raised concerns about the time and travel considerations of getting vaccinated, and others identified distance to the nearest vaccination clinic or hub as a barrier.

Factors relating to COVID-19 vaccination intention reflected the experiences of the different barriers and enablers to COVID-19 vaccine uptake. One factor was delaying the decision to be vaccinated in response to information overwhelm and concern about vaccine safety. For example, some interviewees indicated that they were in no rush to be vaccinated, while others indicated that they preferred to wait rather than get the vaccine. When asked what would change that preference, responses ranged from interviewees feeling they needed more time to decide or more information to support their decision. The other factor was waiting (but wanting) to be vaccinated—these interviewees believed in the safety and efficacy of the vaccine and saw clear personal and social benefits if they could be vaccinated. There was a lack of clarity about eligibility among the interviewees. There were also several accounts of vaccine appointments being cancelled or rejected because local health clinics did not have sufficient supply. These issues relate to the barriers around access and indicated a willingness of individuals to be vaccinated but a failure of health systems and government policies to support that.

The key observations of the interviews are summarized in Table 4.

## 4. Discussion

The aim of the study was to investigate the factors affecting COVID-19 vaccine uptake among regional and rural women aged 30–44 in Australia needs.

### 4.1. Key Determinants of Vaccine Uptake

The study found that information sources about COVID-19 vaccination were a significant factor affecting vaccine intention, acting as both a barrier and enabler to COVID-19 vaccine uptake among younger rural women. In particular, the sheer volume of information about COVID-19 vaccination created mistrust among younger rural women overall. The consequence of this was a desire for more contextualized or localized information, or avoidance of the topic altogether. This sometimes created an opening for family and friends to have a greater influence on understanding of the vaccine, which could lead to more confusion or exposure to misinformation—this has been shown elsewhere to influence vaccination intention negatively [32]. The experiences of younger rural women were found to reflect existing research into the impacts of confusing information which shows that frequent exposure to contradictory health advice on the same issue leads to overall doubt, including in official public health recommendations [33]. This explains how even individuals with high levels of vaccine literacy may not feel adequately equipped to make a decision about being vaccinated against COVID-19. Importantly, this finding also supports the theory that the coronavirus pandemic is also an infodemic, fueled by confusing and inaccurate information [34].

The study identified that access to the COVID-19 vaccine is another key factor affecting vaccine uptake among younger rural women in Australia. Access was found to be an issue for most participants, closely linked to awareness and a need for clearer information about eligibility and booking processes. The study also found that even once a booking was made, other logistical barriers—such as childcare and distance to clinic—could make vaccination an inconvenient activity. The consistent identification of access issues indicates that there are structural inequities affecting younger rural women that may be shaping COVID-19 vaccine uptake negatively. This reflects existing research into rural health inequality—rural health patients tend to be more likely to be uninsured or underinsured, carry higher levels of chronic diseases and higher overall mortality rates [18,19] which makes them more vulnerable to any COVID-19 disease outbreaks. Furthermore, any patterns of spatial inequality in Australia cannot be adequately understood without considering gender [35], reiterating that the intersection of gender and rurality has the potential to deepen inequality of access.

The study also generated valuable insights into individual perceptions of COVID-19 vaccination among younger rural women, and how these factors positively and negatively affect COVID-19 vaccine uptake. Many participants indicated negative views towards COVID-19 vaccine safety and effectiveness, particularly based on concern around the speed of the vaccine’s development. Yet most participants had high support for vaccinations more generally. The study also provided deeper insight into perceptions of the COVID-19 disease, with many participants having little personal concern for themselves catching COVID-19. Therefore, the study identified that the health benefits of vaccination were not a motivating factor for getting vaccinated because the impact of COVID-19 disease outbreaks on regional and rural communities had been so small. Instead, personal benefits (such as greater freedoms) were more important factors in influencing vaccination intention. These findings affirmed other research identifying the greater levels of concern among younger women towards the COVID-19 vaccine’s safety, clarifying that this concern matters in rural and regional settings as well [12]. This lower level of acceptance might be explained by low vaccine confidence, also identified in the study, which has been found to be a strong predictor of low vaccination uptake for COVID-19 [36].

### 4.2. Recommendations

Reaching COVID-19 vaccination targets is not only important for Australia in 2021 but will continue to be an ongoing health challenge for the nation, since ensuring that vaccine uptake is as high and equitable as possible is essential to ensuring that health systems are able to manage, and lives are saved.

Addressing misinformation and disinformation related to COVID-19 vaccination through improved risk communication practices is crucial to be able to ensure that all communities and populations have the information they need to get vaccinated [37]—this should start with more aligned messaging from the Australian Government and relevant state and territory health departments. Engaging peer experts among younger rural women would also help to support stronger trust through the synthesis of official messages into common language and settings [15]. Given that the information source of television was found to be a strong predictive factor for COVID-19 vaccination intentions, it should be included as a key channel in any public health campaigns targeting younger rural women. Information about the COVID-19 vaccine for younger rural women should also address pregnant and breastfeeding women, as well as speaking to rural circumstances. Given that ongoing vaccine rollout will also include younger age groups, information should also address vaccination for children in rural areas, given the important decision-making role that the study’s cohort could play in children’s vaccine uptake. Communication should also consider multi-directional rather than one-way engagement, applying methods of social listening to ensure that there are formal spaces for airing and understanding concerns, since the potential for community feedback to inform ongoing improvement of risk communication strategies is significant [2]. Implementing these recommendations would proactively address the challenges that an abundant—and sometimes overwhelming—information environment can create for those considering vaccination and would specifically help to counter some of the barriers around understanding and access that were identified in the study.

Another important theme that emerged in the study was the importance of tackling complacency, known to be a contributing factor to vaccine hesitancy [38]. The COVID-19 disease was perceived as carrying a low risk within the target population, driven by the lower population densities and lower instances of outbreaks in rural and regional settings. Therefore, information about COVID-19 vaccination for younger rural women should not focus on perceived threats of the disease but should place greater emphasis on the personal benefits of vaccination, including reduced social restrictions and increased freedom to travel domestically and internationally. The study’s findings suggest that these messages will resonate more with younger rural women and may provide greater incentive to be vaccinated than messages that focus on the health benefits.

The study also found that social determinants of health played an important role in shaping COVID-19 vaccine uptake in the target population. The negative impacts of the COVID-19 disease are partly explained by inequities of health access, and the structural barriers that access imposes for those who may not have the time, money, or knowledge to seek out health services such as vaccination [39]. The study has demonstrated that access barriers to COVID-19 vaccination must be addressed if adequate vaccine uptake is to be achieved. The supply issues that have shaped Australia’s COVID-19 vaccine rollout are an important factor to be considered [21], however these issues also reflect greater rural health inequities created by distance and affordability. Addressing these structural inequities at an immediate and longer-term level is crucial—current evidence suggests that COVID-19 disease outbreaks will become endemic [40], meaning that health system vulnerabilities in regional and rural communities will only become more exposed or exhausted over time. Therefore, the design of ongoing and future COVID-19 vaccine rollout strategies should prioritize access and affordability for the target population. This should include consideration of more proximate and convenient vaccination clinics, while also addressing concerns around time and childcare that younger rural women noted as barriers in the study.

### 4.3. Limitations

A limitation of this study was that it focused on a specific population, and this justified the narrow sample choice. However, the use of convenience sampling in the questionnaire (which informed the purposive sampling in the interviews) meant that the sample was not as large or as representative as it could have been. Therefore, the results must be interpreted with this in mind. The sample size limited the statistical analyses which could be conducted, though this was partially balanced through the mixed methods design. Additionally, the limitation of a cross-sectional survey is that it provides an insight into a point in time only, which might not necessarily be indicative of future behaviors, and which limits the ability to establish causality. Furthermore, the data was collected over a two-month period when significant policy shifts around COVID-19 disease control and vaccination occurred (including statewide lockdowns and changes to vaccination eligibility), meaning that responses were made in differing circumstances, reducing the comparability of some data.

## 5. Conclusions

The study has found that in order to achieve high and equitable COVID-19 vaccine uptake, governments, policymakers and health professionals must take into consideration the individual and structural factors affecting younger rural women living in Australia. The study addressed a gap in the research to provide deeper insights into those factors—both barriers and enablers—and how they might be addressed to increase COVID-19 vaccine uptake.

The study has highlighted several important insights and lessons for improving COVID-19 vaccine uptake among younger rural women, especially in higher income countries. It has shown the need to provide clear, contextualized information sources that motivate the individual in their decision-making process. It has also identified the importance of tackling complacency towards the disease by focusing on the individual benefits of vaccination such as increased freedom to travel, as well as making it easier, clearer and more convenient to book and receive a COVID-19 vaccine.

As COVID-19 vaccine rollout continues globally, understanding factors affecting vaccine uptake across a range of communities, such as younger rural women, has important consequences. The lower levels of willingness to accept a COVID-19 vaccine among younger women have not only been identified in Australia but in other countries too. Studying younger women in rural and regional settings adds an important layer to other findings within the global field of rural health research, since these settings already experience health inequities that may compound the consequences of low COVID-19 vaccine uptake or further disease outbreaks.

Further research among younger rural women should be conducted to understand COVID-19 vaccination behavior and coverage, particularly as COVID-19 disease management shifts from a pandemic to an endemic approach and individuals become eligible for further COVID-19 vaccines. Further research should also be conducted to address any fertility or child-related concerns within this demographic that may affect COVID-19 vaccine uptake. Ensuring that the COVID-19 vaccine rollout is equitable and evidence-based—grounded in insights from populations that might display higher levels of vaccine hesitancy, such as younger rural women—continues to be essential to achieving the levels of coverage needed to manage the COVID-19 pandemic globally.

## Figures and Tables

**Table 1:** Sociodemographic characteristics of the questionnaire respondents.

Characteristic		Number
Geography	Major City (Excluding Capitals)	22 (24.4%)
	Inner Regional	34 (37.8%)
	Outer Regional	30 (33.3%)
	Remote	1 (1.1%)
	Very Remote	3 (3.3%)
Education	Postgraduate degree or above	28 (31.1%)
	Bachelor’s degree	37 (41.1%)
	Diploma or Certificate	17 (18.9%)
	Up to Year 12	7 (7.8%)
	Up to Year 10	1 (1.1%)
Occupation	Employed	56 (62.2%)
	Healthcare professional	12 (13.3%)
	Stay-at-home parent	16 (17.8%)
	Student	6 (6.7%)
Pregnant	No	82 (91.1%)
	Yes	8 (8.9%)
Children	0 children	34 (37.8%)
	1+ children	56 (62.2%)

**Table 2:** Summarized findings on vaccine acceptance among younger rural women.

Variable		Number
COVID-19 vaccine acceptance		
Do you intend to be vaccinated against COVID-19?	No	16 (20.2%)
	Uncertain	19 (24.1%)
	Yes	44 (55.7%)
Do you think the vaccines developed so far are safe?	No	9 (11.4%)
	Uncertain	37 (46.8%)
	Yes	33 (41.8%)
Do you think the vaccines developed so far are effective?	No	8 (10.1%)
	Uncertain	39 (49.4%)
	Yes	32 (40.5%)
Should vaccination against COVID-19 be made mandatory for everyone?	No	34 (43%)
	Uncertain	25 (31.6%)
	Yes	20 (25.4%)
Should vaccination against COVID-19 be made mandatory for the most at-risk groups?	No	27 (34.2%)
	Uncertain	15 (19%)
	Yes	37 (46.8%)
Should children be vaccinated too?	No	21 (26.6%)
	Uncertain	31 (39.2%)
	Yes	27 (34.2%)
General vaccine acceptance		
Have you ever been vaccinated against the flu?	No	17 (21.5%)
	Yes	62 (78.5%)
Have you ever wanted to be vaccinated against the flu but couldn’t because you weren’t able to access a vaccine?	No	72 (91.1%)
	Yes	7 (8.9%)
How much do you agree with the following statement: “I am not in favour of vaccines because they are unsafe.”	Disagree	66 (83.5%)
	Uncertain	9 (11.4%)
	Agree	4 (5.1%)
How much do you agree with the following statement: “There is no need to vaccinate because natural immunity exists.”	Disagree	66 (83.5%)
	Uncertain	10 (12.7%)
	Agree	3 (3.8%)

**Table 3:** Significant associations with intention to be vaccinated against COVID-19.

Independent Variable	Degrees of Freedom	Sample Size	Significant Association (*p*, Chi-Squared)	Effect Size (Cramer’s V)
Vaccine acceptance				
COVID-19 vaccination to include children	4	79	<0.001	0.45 (strong)
Perceived benefits of COVID-19 vaccination				
Easier domestic travel	4	79	<0.001	0.45 (strong)
Lifted social restrictions	4	79	<0.001	0.36 (strong)
Easier international travel	4	79	0.004	0.31 (strong)
Information sources on COVID-19 vaccination				
Online news	2	79	0.004	0.37 (strong)
Television	2	79	0.029	0.30 (moderate)
Government websites	2	79	0.040	0.29 (moderate)
Preventive health behaviours against COVID-19				
Wearing a mask	2	79	0.012	0.34 (strong)
Getting influenza vaccine	2	79	0.020	0.31 (moderate)
Social distancing	2	79	0.048	0.28 (moderate)

**Table 4:** Key observations of the interviews.

Category	Factors Affecting Vaccine Uptake *(Italicised Factors Shown in Comments)*	Examples of Participant Comments
Information sources and vaccine literacy	Low Trust in Available Information; *Overwhelmed or Confused by Information*; *Avoiding or Not Actively Seeking Vaccination Information*; Needed More Specific and Clear Information Related To Their Circumstances; High Trust in Scientific Information; High Trust in Government Information; Family and Friends as Sources of Information; High Trust in Information from Health Professionals	“(The news), it’s a bit too much for me. It’s all a bit too noisy. I don’t know how to filter through it.”—Interviewee 8
		“To be quite honest, I haven’t done a lot of my own research, purely to eliminate my fear.”—Interviewee 7
Vaccine acceptance	*Concerns Around COVID-19 Vaccine Safety*; Concerns Around COVID-19 Vaccine Effectiveness; Some Hesitation about Vaccination Generally; Getting COVID-19 Vaccine Feels Forced; Support Vaccination in General; COVID-19 Vaccine Is Safe and Effective; *Not Personally Concerned About Catching Covid-19*; Concerns about the Impact of the Disease; Chronic Illness Increases Concerns about Risk of Disease or Vaccine	“It makes me nervous thinking, well, this vaccine has been around for a year… It’s like there are too many unknowns with it that make me feel that I don’t want to rush out and get it. Not saying I wouldn’t get it, but it makes me uncomfortable to. I wouldn’t be the first in line.”—Interviewee 5
		“Where we live, it seems like COVID has never really existed because we haven’t had any cases yet. It’s sort of a mythical creature at the moment.”—Interviewee 7
Perceptions of trust and vaccine benefits	*Getting Vaccinated Has Individual Benefits*; Getting Vaccinated Helps Others; Trust Government Approach to Managing Situation; Concerned about the Vaccine Rollout; Concerned about Impact of Social Restrictions	“I think the benefits of getting the vaccine would mean the freedom of being able to kind of go places and do things that we are kind of limited to … Socially, it means going places and travelling with peace of mind.”—Interviewee 10
Vaccine access	*Unsure about How to Be Vaccinated*; Getting Vaccinated Involves Logistical Barriers	“I’ve actually had a bit of confusion ‘cause I was looking at getting my first shot and there was a bit of confusion of where to actually access it … So I googled it. Got onto one website, rang one pharmacy, also rang one GP clinic but they didn’t have any in stock … neither Pfizer nor AstraZeneca. Since I was not in the high risk category, they said they just didn’t have any on hand … They really had enough left [only] for people in the high risk categories.”—Interviewee 9
Vaccination intention	Delaying Decision to Be Vaccinated; *Waiting (But Wanting) to Be Vaccinated*	“They sent a message saying—we’ve shipped your vaccine off to Sydney and you can’t have your appointment anymore. So the possibility of me getting one, when I’m just not a priority at the moment at all for getting the vaccine, I think that’s pretty s***.”—Interviewee 10
